# Alcohol and Digestion

**Published:** 1905-03-25

**Authors:** 


### ALCOHOL AND DIGESTION.

In the compendious indictment of alcohol recently presented at the Mansion House one of the counts advanced by a distinguished surgeon and physiologist was to the effect that the use of alcohol with food was to be condemned on the ground that it delayed digestion. Allowing that this practice increased the flow of the gastric juice, it was urged that there was a further consequence in the shape of a slowing of the digestive processes, and that as these two results balanced one another, there was no net gain or advantage. This argument obviously assumes the accuracy of the generally-accepted creed that rapidity of digestion is an end to be desired as profitable to the welfare of the entire organism. It must, however, be remembered that this doctrine is open to question. Rapidity of digestion is doubtless accompanied by rapidity of absorption—that is, by a correspondingly prompt introduction of relatively large quantities of nutrient material into the blood-stream, whereas the opposite conditions of digestion and absorption mean a more gradual and prolonged supply of nutriment to the circulating fluid. It must be admitted that the latter state of matters is at least possibly more favourable to even and sustained nutrition of the tissues than the former. Alternating periods of abundant and scanty food supply cannot be commended as ideal physiological conditions, and they are equally open to objection on economic grounds. Whilst it is an obvious advantage that food shall be introduced into the stomach in relatively large quantities at occasional intervals, it is probably well that an even and not an irregular flow shall reach the tissues. Hence the spreading of the digestive process over a comparatively long period cannot be condemned offhand as a physiological disaster. Indeed it may be advanced as the normal condition of healthy tissue nutrition. Some years ago the late Sir Wm. Roberts showed that a number of condiments and beverages habitually used by mankind delay digestion, and he explained their use as helping to neutralise the too rapid absorption apt to result from the practice of cooking to which civilised man subjects most of his foodstuffs. Hence, whatever other condemnations may rest upon alcohol, the fact that it delays digestion may be to its credit rather than otherwise.

